# Insights into the plant response to nematode invasion and modulation of host defense by plant parasitic nematode

**DOI:** 10.3389/fmicb.2024.1482789

**Published:** 2024-11-18

**Authors:** Xiaolong Chen, Fuqiang Li, Ding Wang, Liqun Cai

**Affiliations:** College of Resources and Environmental Sciences, Gansu Agricultural University, Lanzhou, China

**Keywords:** molecular mechanism, plant defense, nematode, signaling, resistance

## Abstract

Plant pathogens cause diseases by suppressing plant immune response and interacting with plant cells. Investigating these interactions assists in decoding the molecular strategies the pathogen uses to overcome plant immunity. Among plant pathogens, the nematodes parasitizing various plants incur a profound impact on food production across the globe. To deal with these parasites, plants have developed a complicated defense system, including performed defenses like rigid cell walls and reinforcements acting as the first line of defense to combat any invader. Plants also have a wide diversity of constitutively released phytochemicals that are toxic to the invading microbes as their defense arsenals. Additionally, a substantial system of host responses is triggered in response to infection based on the abilities of the host plants to sense and recognize the invading pathogen. Nematodes have evolved the strategies to perceive and respond to host defense through their nervous system which help them escape, avoid, or neutralize the host plant defense systems. For developing an effective management strategy, it is crucial to understand the mechanism by which the nematode suppress the host defense. Previous reviews mainly discussed the interaction of plants with the nematodes for their immunity against nematodes. The present review will discuss the strategies employed by the plant parasitic nematodes for suppressing plant defense along with an overall insights into the basic nematode recognition mechanism and basal immunity response of the host plant. The mechanism of modulating host defense by nematodes including the role of their effectors were also discussed. The latest research progress about the release of metabolites by plants, and the mode of action of these defensive chemicals at the molecular level in combating the nematode invasion was also analyzed.

## 1 Introduction

The most widely distributed and omnipresent metazoans on Earth are nematodes commonly found in terrestrial, marine, and freshwater environments (Norton and Niblack, 2020). A great majority of these nematodes rely for their survival on bacteria and various microorganisms. Due to their important position in the food web, they play a vital role in cycling the nutrients in the ecosystem. Nevertheless, these parasites have earned a bad name due to a fraction of the notorious nematodes that cause infections in humans persistently (Qing and Bert, 2019). Similarly, in agricultural farming, a handful of the nematodes parasitizing various plants incur a profound impact on food production across the globe. Management of nematodes, inducing diseases in plants, was done for decades by fumigating the infected field soil or applying different types of nematicides (Mandal et al., 2021). Significant yield losses are induced by these plant parasitic nematodes (PPNs) to important crops. Most crop plants are parasitized by these soil-borne microscopic organisms, affecting these economically important crops and resulting in average severe yield losses as high as 12.3% (Zhao et al., 2020). According to an estimate, worldwide losses induced by these nematodes are worth $80 billion each year, and the exact actual figure might be even higher than this average estimate (Singh et al., 2015).

More than 4,000 plant parasitic nematodes that are obligate biotrophs have been reported feeding on different plant tissues, including leaves, stems, roots, and flowers; however, most of the reported species of PPNs feed primarily on roots (Pulavarty et al., 2021). Based on feeding habits, these PPNs are broadly divided into two groups: the endoparasites and the ectoparasites. The ectoparasitic nematodes get nourishment from the host plants without entering into the plants through their stylet while remaining on the root surface. The food is withdrawn from different types of cells or tissues, depending on the stylet length, including epidermal cells, cortex, and vascular tissues, or simply from the root hairs. They remain on the feed site for some time and then move on to new sites for feeding. Due to their lifestyle, minor damage is induced by a single ectoparasitic nematode when compared with nematodes that are endoparasites. Contrary to ectoparasites, the nematodes that are migratory endoparasites enter the host completely and move through various layers of host tissues, damaging different cells while penetrating the host. The host’s cell wall is first damaged, and the nematode releases saliva into the cytoplasm and then feeds on it. After feeding, the nematode further pierces the cell wall with the help of its stylet and enters the cell, thereby damaging it. The most economically significant plant parasitic nematodes are a small group of endoparasitic sedentary nematodes, including *Meloidogyne* spp. (the root-knot nematodes) and *Globodera* and *Heterodera* spp. (cyst nematodes). In the second stage, infective juveniles (J2s) of these nematodes penetrate the roots of the host plants and enter host cells one by one, damaging the host tissue. A specific hypertrophic and hypermetabolic long-term feeding structure is induced by J2s at the vascular cylinder to procure nutrients from it (Perrine-Walker, 2019).

To deal with these parasites, plants have also developed a complicated defense system, including performed defenses like rigid cell walls and reinforcements acting as the first line of defense to combat any invader. Plants also have a wide diversity of constitutively released phytochemicals that are toxic to the invading microbes as their defense arsenals. Additionally, a substantial system of host responses is triggered in response to infection based on the abilities of the host plants to sense and recognize the invading pathogen (Desmedt et al., 2020). A two-tiered recognition system at the molecular level in host plants evolved to identify the invading pathogens. These include the localized cell surface pathogen recognition system named PRRs (pattern recognition receptors) and MAMPs or PAMPs (microbial or pathogen-associated molecular patterns). The second system is conserved evolutionarily, performing vital functions across a class of organisms during the life cycle. MAMPs or PAMPs induce defense responses of the host through a cascade of complicated signals (pattern triggered immunity, PTI or PAMPs triggered immunity) (Przybylska and Obrępalska-Stęplowska, 2020).

Virulent pathogens overcome these host defenses by releasing compounds called effectors into the host plant. These effectors interfered with PTI responses, resulting in ETS (effector-triggered susceptibility). Host plants may also identify these effectors with intracellular NLRs (nucleotide-binding leucine-rich repeat protein receptors). More effective and robust defense responses (ETI, effector-triggered immunity) are triggered by NLR activation that terminates as programmed cell death, also called HR (hypersensitive response) (Yuan et al., 2021). Generally, plants are immobile and exploit a variety of host defenses involving both structural components as well as chemical compounds. Plant parasitic nematodes evolved various strategies to perceive and respond to these host plant defenses through their nervous system which help them to escape, avoid, or neutralize the host plant defense systems. We explored the information on modulation of host defenses by nematodes employing diverse approaches to host defense suppression. Overall, this review highlights the nematode-plant interactions, the understanding of which can assist in formulating effective management methods. The insights provided in this review serve as a comprehensive resource guiding future studies in host-nematode interactions.

## 2 Plant recognition mechanism for nematode detection

**Figure 1:**
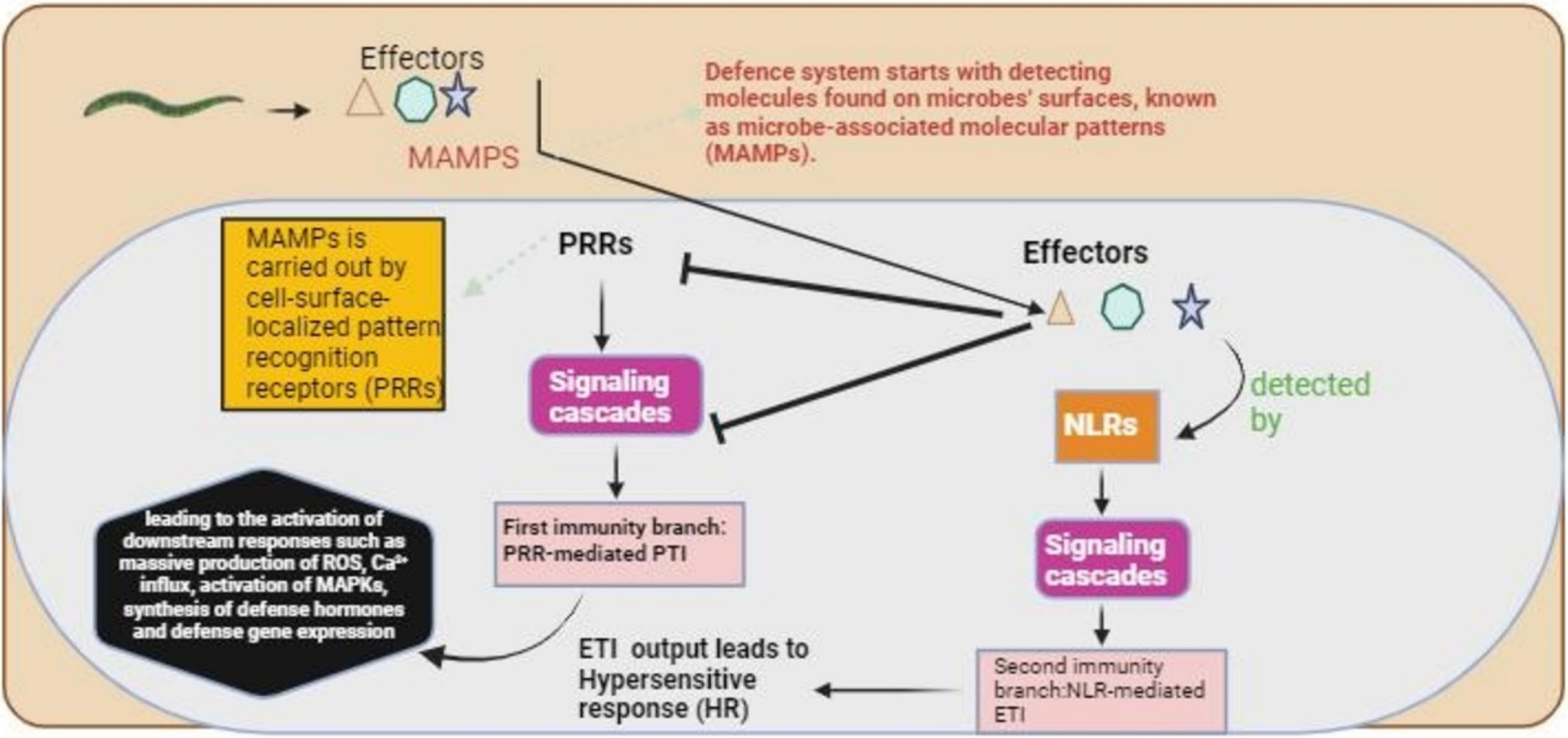
The plant’s basic recognition mechanism for nematode detection. The defense mechanism starts with detecting microbe-associated molecular patterns (MAMPs), followed by the activation of complex signaling cascades, which initiate pattern-triggered immunity (PTI). The effectors produced by the nematodes suppressed the PTI, and these effectors were detected by NLRs, which started a second signaling cascade for the activation of effector-triggered immunity (ETI).

Host plants have the potential to identify and react to DAMPs and PAMPs (Figure 1). Though it was known that the PPNs trigger these responses, the actual machinery of recognition and the patterns involved remained obscure. The host plants detect the PPNs or their actions using special specified protein receptors in the apoplast within the cell. Extracellular kinases and receptor-like proteins survey the apoplastic area of the host plant for special molecular patterns related to infections induced by nematodes (Choi and Klessig, 2016). The receptor proteins have multiple domains comprising an LRR (leucine-rich repeat) domain attached through a transmembrane domain with a plasma membrane. The cytoplasmic kinase domain is also on receptor-like kinases and is substituted with a small tail region (nondescriptive) in the receptor-like proteins (Teixeira et al., 2016). Both the specific effector-induced immunity and the PAMP-induced immunity are activated by the extracellular receptor-like kinases/proteins; however, to date, it has only been revealed to modulate the strong resistances against cyst nematodes (De Lorenzo and Cervone, 2022).

It has been reported that most of the characteristic conventional PAMPs, the Ascarosides, are present in a group of small molecules in the PPNs. These Ascarosides are an evolutionary conserved group of pheromones released by the nematodes that may trigger various classical host defenses related to the perception of PAMP in mono and dicot plants (Cohen and Troemel, 2015). The most widely released ascaroside in plant parasitic nematodes, Ascr#18, induces a classical immune reaction in host plants like gene expression of PTI-marker, mitogen-triggered kinases, and jasmonic as well as salicylic acid modulated signaling mechanisms of host defense. It is important to note that Ascr#18 treatment enhances the resistance levels against cysts and root-knot nematodes in *Arabidopsis*. Furthermore, Ascr#18 is relatively conserved in monocotyledonous and dicotyledonous plants (Klessig et al., 2019). Nevertheless, the analogous PRR for comprehending Ascr#18 has yet to be identified.

Leucine-rich repeat-RLK expressed by Arabidopsis Nilr1 (LRR-RLK I triggered by nematode) is the first recognized PRR that is implicated in the PTI induction against PPN-derived molecule (Nie et al., 2022). NILR1 was secluded as a vital component for identifying the “Nema Water,” a hydrated solution that is incubated along with J2s of RKN (*Meloidogyne incognita*) and CN (*Heteroder schachtii*) as an inducer of PTI. Importantly, the NILR1 extracellular receptors are extensively conserved both in monocots and dicots. This is further verified by the fact that immune response is triggered in sugar beets, tomatoes, rice, and tobacco by Nema Water. Nevertheless, the NILR1-recognized corresponding molecules of PAMP have not been characterized. Though the corresponding nematode pheromone receptors of the host plant remain obscure, the classical PRRs (pattern recognition receptors) are known to play a role in the recognition of PPNs. It has been demonstrated that few protein-based molecules (not Ascarosides certainly) released by the J2s of *H. sachtii* also induce general PAMP recognition reactions, which are BAK1 (BRASSINOSTEROID INSENSITIVE-1 (BRI1)-ASSOCIATED KINASE 1) dependent (Mendy et al., 2017). It is a coreceptor commonly needed for identifying various PAMPs.

**Figure 2:**
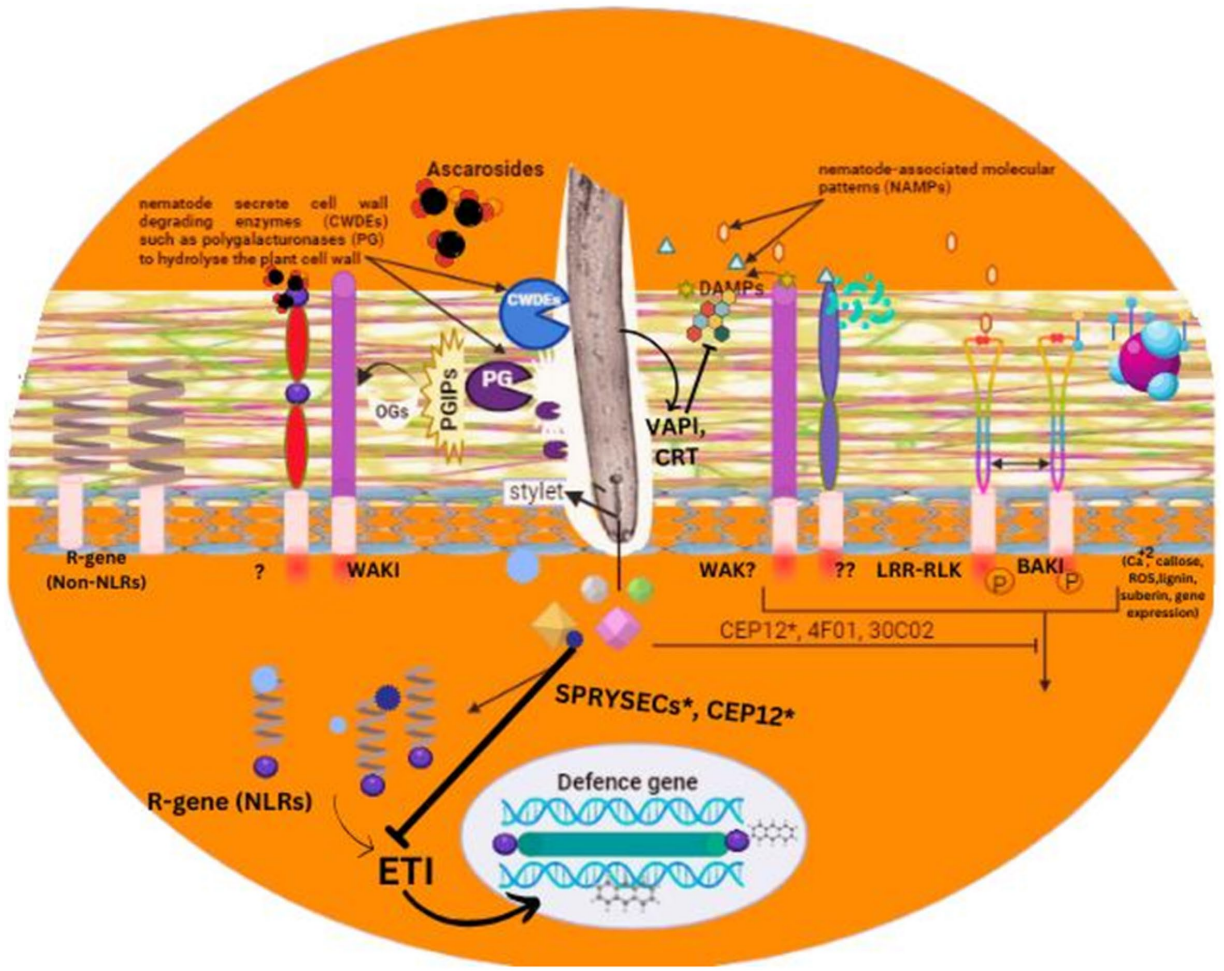
An overview of pathogen associated molecular patterns and their role in plant recognition process for nematode detection.

Moreover, it has also been demonstrated that the LRR-RLKs (leucine-rich repeat receptor-like kinases) expression was triggered in roots when treated with exudates of nematodes, including NILR2 (nematode-induced LRR-RLK 1) required for protein-based PAMP reaction that remains obscured. Considering that the NILR1 has a protein-based ligand, one can speculate that the host plants identify the nematode patterns in at least two ways. In agreement with this notion, it has been demonstrated that the RKN trigger pattern induced immunity in the roots of *Arabidopsis thaliana* in BAK1-independent and BAK1-dependent manners (Figure 2). The NLR proteins play a crucial role in the identification of the PPNs (Peng and Kaloshian, 2014). Kaloshian and Teixeira (2019) reported that NLR proteins employed in plant parasitic nematode recognition are often expressed through the resistance genes (R). The well-characterized R genes are pepper *CaMi* and prune *Ma*; potato *Gpa2* and *Gro1–4*; and tomato *Mi-1.2*, *Mi-9*, and *Hero-A* (Claverie et al., 2011).

R-genes *Ma, CaMi, Mi-1.2*, and *Mi-9* offer resistance against root-knot nematodes, whereas Hero-A, Grol-4, and Gpa2 confer resistance against cyst nematodes. Ma and Grol-4 express TIR-NLRs, while the others express CC-NLRs. Interestingly, the protein encoded by Ma has an extensive and quite polymorphic C-terminal LRR region, which is believed to be vital for identifying plant parasitic nematodes (Claverie et al., 2011). Examples of avirulence factors of PPN identified by NLRs have been identified. Cyst nematode (*Globodera pallida*) secreted SP1a and RYanodine receptor (SPRY) domain (SPRYSEC) proteins Gp RBP-1, acts as an effector that triggers cell death (Hypersensitive responses) along with Ran GTPase-activating protein 2 (RanGAP2) and GPA2 (Sacco et al., 2009). In the Gp-RBP SPRY domain, at position 187, the proline residue is vital for the identification by GPA2. At the same time, a mutation in the Gp-RBP-1 allele of the virulent variant at this position helps it escape host recognition. Host plants recognize intracellular apprehension induced by effectors from RKNs and CNs along with NB-LRR (nucleotide-binding leucine-rich repeat) receptors for immunity (Diaz-Granados et al., 2016).

A central NB domain in all the NB-LRR immune receptors is commonly linked with a carboxy-terminal LRR domain. However, subsequent subdivision is based on their amino-terminal domain, comprising either a TIR (Toll-interleukin receptor-like domain) or CC (coiled-coil domain). In the NB-LRR immune receptor, a specific amino terminus domain is unrelated to a particular feeding type structure or nematode species (Heidrich et al., 2012). As an example, both Gpa2 [CC-NB-LRR] and Grol-4 [TIR-NB-LRR] immune receptors modulate the resistance to CNs of potato, while Mi-1 [CC-NB-LRR] modulates the resistance to RKNs (Xiao et al., 2021). In tomatoes, numerous immune receptors (CC-NB-LRR) have elongated amino terminals believed to be peculiar to solanaceous crops [Hero and Mi-1], regulating the immune receptors function (Warmerdam et al., 2020). Another remarkable discovery is the latest WRKY-like domain identification of the *Ma* immune receptor’s carboxy-terminal to RKNs in Prunus [TIR-NB-LRR-WRKY] (Rinerson et al., 2015). The ETI and defense-related gene expression in PAMP is regulated by the correlation of the transcription factor of WRKY (Eulgem and Somssich, 2007). In Ma, the WRKY-like domain presence may suggest the integration of the potential to identify invaders with direct modulation of the expression of defense genes. On the other hand, the WRKY-like domain also appears like an RKN virulence target due to its proximity to the domain TIR-NB-LRR of Ma. This auxiliary domain behaves like bait or decoy (Warmerdam et al., 2020). Recently, another study revealed that the transcription factor TCP9 of *Arabidopsis thaliana* modulates the root system architectural plasticity to *H. schachtii* invasion through ROS-mediation (Willig et al., 2022).

## 3 Basal immunity response to nematode

**Figure 3:**
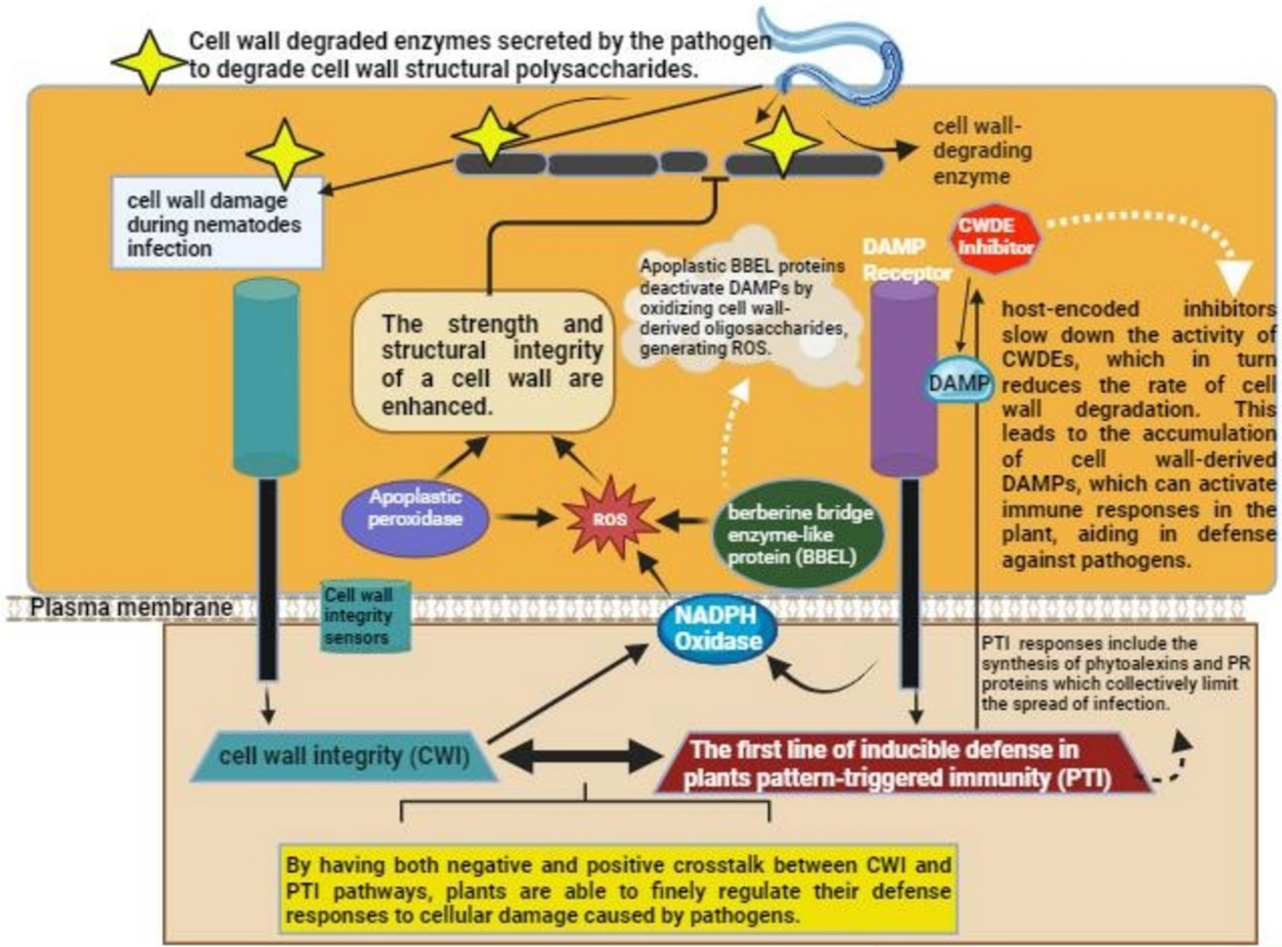
Mechanism of the activation of Plant immunity upon nematode/pathogen invasion. Nematodes secrete cell wall degrading enzymes that degrade cell wall of the host. DAMP, Damage associated molecular pattern; ROS, reactive oxygen species.

Plants exploit a multilayered intrinsic immune system to help themselves defend against various parasites and pathogens, which depends mainly on recognizing specific molecular patterns distinctively related to infections. The initial inducible defense line of plants-basal defense systems- is triggered through extracellular recognition pattern receptors (Saijo et al., 2018). DAMPs are recognized by these receptors, which are usually the fragments of cell walls produced as a result of invasion of roots of the plants or PAMPs that are generated directly from virulent pathogens (Cicchinelli et al., 2024). This recognition triggers a PTI-like defense response. It is, however, still not clearly observed whether the PAMPs or DAMPs-based induction occurs during the invasion process by nematodes and whether it carries any significance of host susceptibility against nematodes (De Lorenzo and Cervone, 2022). In plants, basal defense involves a range of quick chemical responses, such as the production of antimicrobial secondary metabolites, reactive oxygen species, protease inhibitors, hydrolytic enzymes, and the strengthening of the cell walls of the plants through callose deposition and lignification (Li et al., 2023) (Figure 3).

The migration and invasion stages of *H. glycines* infecting Arabidopsis, lasting approximately twice the same in *H. schachtii*, correlate with excessive callose deposition and cell necrosis on the migratory belt (Sangi et al., 2023). The necrosis produced is not because of any mechanical destruction by the migratory nematode; instead, it is the result of hydrogen peroxide liberation. The analysis of the nematode-invaded roots gene expression revealed that the basal defense is triggered during the nematode migration and at the developing feeding structures of resistant and susceptible plants. At the same time, it is suppressed within the feeding structures of the susceptible plants. Furthermore, the nematode’s intracellular migration induces stronger basal defenses in host plants compared to the nematode’s intercellular migration within the plants (Holbein et al., 2016).

## 4 Plant strategic response to nematode infection

Several strategies are employed by the plants against nematode invasion, one of which is to utilize the endodermis. The vascular tissues of roots are covered by a sheath of specialized cells known as endodermis. The reinforcement of the cell walls seals the apoplast of the endodermal layer, like deposition of lignin in transverse and radial cell walls, the Casparian layers, and suberin impregnation of the primary cell walls (Eves-van den Akker, 2021). Hence, the nutrients and water-free diffusion across the vascular tissue, both in and out, is blocked, besides hampering the inoculation of some pathogens. Endoparasitic migratory nematodes like *Pratylenchus* spp. obtain their food totally from the cortex, indicating incompetency to penetrate through the endodermis to exploit enormous amounts of food in the vascular bundles (Mathew and Opperman, 2020). On the contrary, the feeding sites of endoparasitic sedentary nematodes like RKN (*Meloidogyne* spp.) are established inside the vascular system (Perrine-Walker, 2019). Nonetheless, these nematodes do not cross the endodermis directly. These nematodes penetrate the roots at the zone of elongation of the root tips, circumventing the barriers offered by endodermis, subsequently migrating through the cortex upto the meristem of the origins that lacks the reinforcement of the cell wall. Finally, they reach the central cylinder through the differentiation zone from the front side (Li et al., 2015). Besides physical blockage, the endodermal layer also affects the nematode development by modifying nutrient and water flow across the feeding sites established by these endoparasitic sedentary nematodes. Notably, the given hypothesis further highlights the important role of the endodermis during the process of nematode invasion of host plants. Plants, upon sensing the infection of nematodes, may enhance lignin deposition, which further increases the strength of the cell wall (Veronico et al., 2018). The available literature also describes the role that lignin plays during nematode-plant interaction. Researchers have revealed that host plant resistance against endoparasitic migratory nematodes is associated with enhanced deposition of lignin in the resistant bananas host plant cell walls. The plants further react to the nematode invasion by increasing cell wall lignification (Mathew and Opperman, 2020).

Upon pathogen infection, wounding, or treatment with PAMP, the enhanced callose deposition between the plasma membrane and the cell wall suggests PTI in plants. Additionally, the plasmodesmata size exclusion limit is modified due to callose deposition, contributing to symplastic transport regulation (Tran et al., 2017). Nevertheless, during the nematode-plant interaction, the role of the injury or pathogen-triggered callose deposition has yet to be explored. The ectoparasitic ring nematode, *Criconemella xenoplax*, feeds primarily on the epidermal cells of the roots. During feeding, when the nematode stylet damages the cell walls, callose-like material is deposited in all the cells between the stylet of the nematode and the cell membrane. The callose deposition results in the stylet encasement in a thick callose layer upon coming in contact with the plasma membrane of the cell, except its aperture, at the site of feeding (Tran et al., 2017).

**Figure 4:**
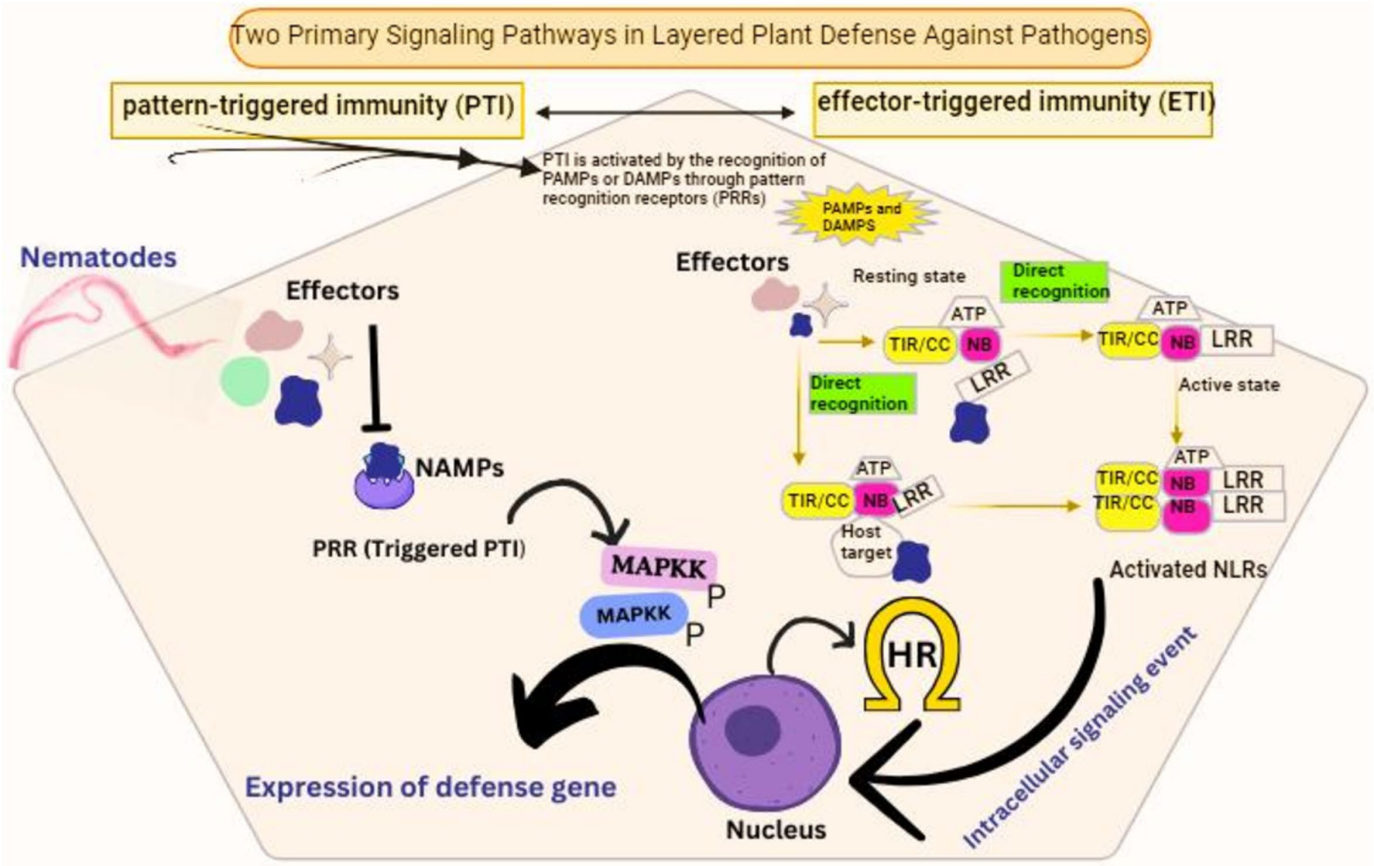
Signaling pathways involved in host plant defense activation against nematode infection. NAMPs, nematode associated molecular patterns.

The deposition of the callose-like material has been exhibited both during incompatible and compatible nematode plant interactions, yet the relationship of these depositions to the susceptibility of the host plant still needs to be better understood. Nonetheless, it has been revealed in a recent study that a reduction in the susceptibility in Arabidopsis was observed on the enhanced expression of the ethylene response transcription factor RAP2.6 against *H. schachtii* accompanied with JA-related genes overexpression and increased deposition of callose at the infection sites produced by the invading nematode (Kammerhofer et al., 2015). In grasses, cereals, and rice, the important role that the deposition of callose plays in the development of resistance against root-knot nematodes has already been demonstrated in connection with lignin and suberin (Ji et al., 2015). Based on these research findings, the deposition of callose can be evoked as the basal defense during the migration of nematodes in the roots of host plants. However, the extent and amount of the callose deposition may fluctuate depending on various factors like the migration pattern of the nematode, the behavior of nematode feeding, and the compatibility of the host (Figure 4). It is in dire need of time to have further insight to help us correlate the callose deposition variation and the host plant susceptibility through innovative genetic, histological, and microscopic tools. It is essential to test the alteration in the line in terms of the deposition of callose and the degradation potential against various species of nematodes (Yang et al., 2024). This insight will be very helpful in establishing the correlation of callose deposition in the basal defense response against different nematodes.

## 5 Molecular mechanism of modulating host defense by nematodes

**Figure 5:**
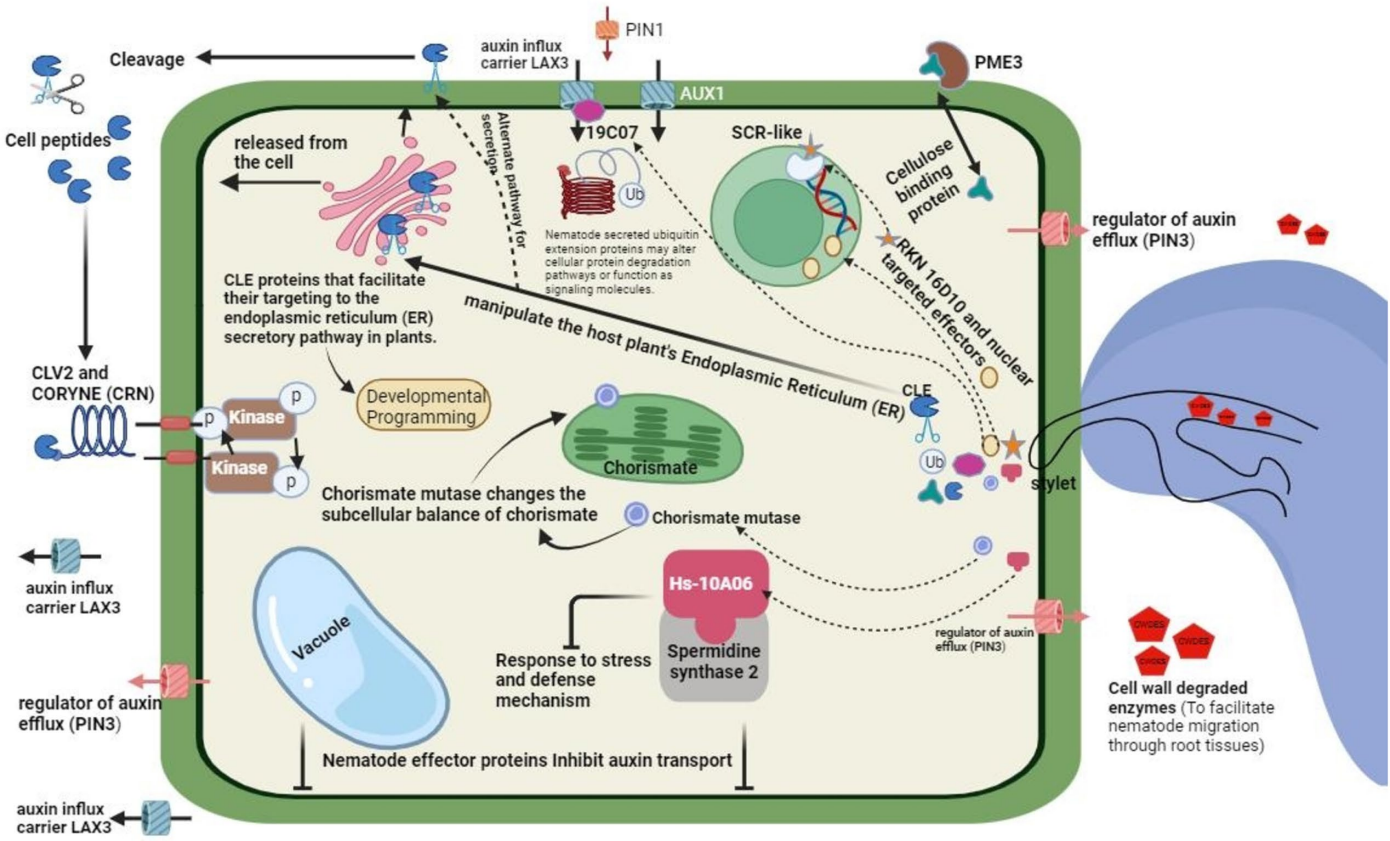
General mechanism of modulating host defense by nematode, role of effector proteins and general invision.

Different PPNs have evolved several complementary pathways to help them protect themselves and their feeding sites against host plant defense responses. PPNs employ three strategies to modulate the host defenses, all of which are not mutually exclusive (Figure 5). In line with the nematodes parasitizing animals, plant-infecting nematodes also employ special means to evade extracellular immune receptor recognition. Furthermore, effectors are delivered into the cytoplasm and the apoplast of the host cells to modulate the signaling of immune pathways (Przybylska and Obrępalska-Stęplowska, 2020). Finally, as a final attempt, they switch back to the detoxification mechanisms and enzymes to overcome the potential detrimental chemical defenses of the host plants. Antioxidant system components are deployed to neutralize the damaging reactive oxygen species released by the host. Reactive oxygen species (ROS), besides the direct killing of the nematodes, can enhance the strength of the cell walls of the hosts through cell wall polymer crosslinking, generating and strengthening the inter and intra-cellular defense signals and modulating the cell death responses linked with the defense responses (Khan and Khan, 2021).

The defense burst of oxidative products of the plants is neutralized by the variety of antioxidant enzymes in the hypodermis and on the surface of the endoparasitic nematodes. The most important and abundant are peroxiredoxins, which neutralize the antioxidant enzymes at the interface of plant and nematode. The nematode species *G. rostochiensis* releases a variant of glutathione peroxidase (Gr-GPx-1) from its hypodermis that detoxifies the plant-released ROS (Ali et al., 2015a,b). Moreover, intact membrane lipid peroxidation is protected by a glutathione peroxidase cuticular homolog of *Brugia pahangi* (filarial nematode) through peroxidized fatty acids transition into their corresponding alcohols. The hypodermis of the endoparasitic nematodes is safeguarded by employing a multilayered strategy of antioxidants against host plant-deduced oxidative stresses (Lin et al., 2016). Initially, peroxiredoxins are used to eliminate hydrogen peroxides in the host plant apoplast, which can reduce their free radical exposure; secondly, through the release of glutathione peroxidases, which help in protecting the exterior of the cell membranes of hypodermis from being affected by these radicals (Li et al., 2016).

Effectors are also released by the Cyst nematodes that have the potential to modulate the cellular components redox state in host plant cells. As an example, the effectors of *H. schachtii* (4F01) most seemingly acts as a regulator of redox through its interaction with an oxidoreductase belonging to the *A. thaliana* 2-oxoglutarate Fe(II)-dependent oxygenase family (Vieira and Gleason, 2019). Infection of nematodes triggers the protein expression related to the pathogenesis, which hampers the reproduction and development of nematodes. Recently, the 30 CO_2_ effector of nematode species *H. schachtii* has been demonstrated to interact physically with the *β*-1,3-endoglucanase, characterized as a PR-2 protein, in the apoplast of *A. thaliana* (Kazan, 2018). A knockout mutant of this *A. thaliana* β-1,3-endoglucanase exhibited an increased susceptibility to infections caused by nematodes, and enhanced expression reduced the number of nematodes that could induce infections. The signaling cascade of extracellular protease modulates the systemic and local defenses against parasites and pathogens in the host plants (Biere and Goverse, 2016).

Lipid peroxidases are vital in the chemical defenses of plants against various pathogens and are important as defense second messenger signaling. PPNs release a particular fatty acid class and FAR (retinol binding proteins), which seemingly intervenes with the host lipid-based defenses (Karanastasi et al., 2018). A mutated FAR protein (Gp-FAR1) produced in the *G. pallida* (cyst nematode) bound to linoleic and linoleic acids that served as precursors to JA signaling rather than jasmonic acid. In host plant immune signaling, intracellular Ca^2+^ acts as a leading mediator. In host plant nematode interactions, signaling of Ca^2+^ is yet to be explored for a complete insight. Nevertheless, the application of La3Cl (Ca^2+^ channel inhibitor) exogenously to the roots of potatoes revealed that the signaling of Ca^2+^ is a restricting factor in the initiation of feeding structure as well as plant invasion by CNs (Manosalva et al., 2015). RKNs release calreticulins, which may intervene with immune signals modulated by Ca2+ in the plant hosts (Wei et al., 2021).

In resorcinol-triggered secretions of the stylet of *M. incognita*, calreticulin Mi-CRT specific to pharyngeal glands was recognized and was later found in the host cell apoplast along with the migratory belt as well as in areas adjacent to the giant feeding cells (Liu et al., 2024). Numerous plant pathogenic microbes intervene with the signaling of host plants’ immune systems by captivating the host plant’s proteasomal degradation system. Two effectors of the *G. rostochiensis* (potato cyst nematode) may for a probable connection between immune suppression and proteasomal degradation in the plant host. Initially, a universal GrUBCEP12 (carboxyl extension protein) specific to the pharyngeal glands of potato cyst nematode (*G. rostochiensis*) represses the defense response of the host plants. Recently characterized effector SPRYSEC present in the stylet secretions of *G. rostochiensis* may repress the host’s defense responses by acting as a flexible modifier of substrate specificity of plant host-driven E3 universal ligase complexes (Mitchum et al., 2013; Vieira and Gleason, 2019). Numerous family members of SPRYSEC of potato CNs restrictively repress HR (programmed cell death defense response) and resistance against diseases modulated by the host immune receptors of the type CC-NB-LRR (Elmore et al., 2011; Chen et al., 2016).

### 5.1 Insights into the mechanism of nematode effectors suppressing host defense

Plant parasitic nematodes produce specific molecules in the host plant interaction space, which is known to help in their capacity to navigate detrimental plant environments. Effector proteins, among such molecules, specifically play vital roles. Various effectors produced by nematodes have been demonstrated to facilitate the repression of host defenses (Vieira and Gleason, 2019). However, for most of these effectors, the modes of action have been figured out, for many additional effectors, it has been only revealed that they can suppress defenses without determining the underlying mode of action. The diversity and extent of pathways used by the nematodes to restrict the host’s defense responses suggest their significance to successful infection. Plant parasitic nematodes utilize most of their genomic efforts to repress the defenses of the host plants (Eves-van den Akker and Birch, 2016). A few genes, like VAPs, are frequently found among various parasitic nematodes of the phylum. Nonetheless, most effectors are new genes present among small groups of PPNs and invade different facets of the host plant defenses/PTI (Davies et al., 2015). As briefed above, only a few cases may enhance host susceptibility against various pathogens. In this case, the suppression of host defense may act as a factor in the triggered susceptibility of the host plants (infected with a nematode) to various pathogens. A detailed and thorough investigation of defense suppressors of nematodes is of utmost importance to provide a better insight into the PTI response of the host plant (Pogorelko et al., 2020).

The parasitizing nematode delivers the effectors around or into the plant cells and tissue through its style, where the defense responses of the host plant are mounted against the invading nematodes (Tran et al., 2017). It is becoming increasingly clear how various mechanisms are employed to target the host plant defenses. It is, however, not surprising that the plant proteins, having clear-cut defense functions, are targeted by the effectors released by the invading nematode. The proteins involved in recognizing the signals and signal transduction are targeted, resulting in the induction of host defense responses (Goode and Mitchum, 2022). The effectors of PPNs regulate proteases and kinases that play a role in signal transduction. Several effectors link and assumingly denature proteins directly responsible for defense response, like a few proteins related to pathogenesis. There are also a large number of research findings revealing the role of effectors in intervening with the incarnation of recognized defense responses such as programmed cell death or other defense trademarks, though without exhibiting the effector’s actual mode of action (Liu and Lam, 2019).

VAPs (secreted venom allergen-like protein) are universal among plant parasitizing nematodes, and their role as effectors suppressing host defenses is well established in cyst nematodes. These VAPs mediate the basal immunity of host plants through selective invasion of the host immune receptors on the surface layer of injured host tissues (Pogorelko et al., 2020). Recently, it has been reported that root-knot nematodes release ligand mimic effectors which attack the FERONIA receptor-like kinase to intervene with the induction of PTI. An exciting example of repression of host defense by effector of *Heterodera* nematode species is HgG1 and 18 (Przybylska and Obrępalska-Stęplowska, 2020). Throughout the nematode parasitic stages of the life cycle, the protein is produced and translocated to the cell nuclei of soybean after the nematode infection. This effector’s persistent and immune solid repressive potential has been reported and most probably was linked with the nucleus-based functions of the nematode effector (Khan and Khan, 2021).

Effector 4E02 of Heterodera is another example of modifying host plant defense responses through the mode of action of the effector (Table 1). The protein of these effectors binds to the papain protease RD21A, a well-known enzyme responsible for regulating the host plant defense responses, specifically in the vacuole of the Arabidopsis (Shi et al., 2018; Anuoluwa et al., 2024). Effector 28B03 of *Heterodera* is another fascinating example of host signal transduction pathway modulation in host defenses. This protein-bound and inhibited the stress-related kinase in *Arabidopsis* specifically that resulted in the destruction of the cascade of kinase and hence syntaxin protein phosphorylation of the host plant, which has been demonstrated to alter the antimicrobial proteins related to pathogenesis in the apoplastic area (De Kesel et al., 2020; Khan et al., 2023).

**Table 1:** Effectors of different plant parasitic nematodes and their action mechanism for modulation of plant defense.

Effector	Nematode	Mechanism	Host	References
Venom allergen-like proteins	CNs	Modify plant resistance by binding host surface receptors related to broken host tissues	*A. thaliana*	Lozano-Torres et al. (2014)
Ligand mimic effectors	RKNs	Interfere pattern triggered immunity of the host by targeting the receptor-like kinase	*A. thaliana*	Zhang et al. (2020)
SPRYSECs	*Globodera*	Modulate the pathways of effector trigger immunity and pattern triggered immunity	*Nicotiana benthamiana*	Ali et al. (2015a,b)
HgGland18	*Heterodera*	Effective in suppressing immunosuppressive ability along with genetic modulation	*N. benthamiana*	Noon et al. (2016)
4E02	*Heterodera*	Blocking of RD21A, an enzyme that regulates plant immunity in *Arabidopsis*	*N. benthamiana*	Pogorelko et al. (2020)
Ubiquitin ligase	Potato CN	Inhibition of host defense by binding defense regulated proteins and enzymes	*N. benthamiana*	Kud et al. (2019)
Annexin-like effectors	Several nematodes	Inhibit host defense by causing death of plant tissue	*Nicotiana benthamiana*	Ali et al. (2015a,b)
30C02	*Heterodera*	Modulating functions of *β*-1,3-endoglucanase, a pathogenesis-related protein, resulting defense inactivation	*Arabidopsis*	Hamamouch et al. (2012)
MjTTL5	RKN	Activation of ROS	*Arabidopsis*	Lin et al. (2016)
Mg01965	*Meloidogyne*	ROS suppression activated by flg22 during apopalast accumulation	*N. benthamiana*	Zhuo et al. (2019)
GLAND5	*Heterodera*	Alteration in the expression of defense genes, callose deposition, and inhibition of ROS	*A. thaliana*	Yang et al. (2019)
MilSE5 and MilSE6	*Meloidogyne*	Suppression of cell death activated by other microbial pathogens	*Arabidopsis*	Shi et al. (2018)
16B09	*Heterodera*	Suppression of flg22-activated gene expression pattern	*Glycine max*	Hu et al. (2019)
MSP18	*Meloidogyne*	Elicitin-induced cell death suppression	*Oryza sativa*	Grossi-de-Sa et al. (2019)
Ha18764	*Heterodera*	Suppression of ROS, cell death, expression of defense related genes, and callose deposition	*A. thaliana*	Zhang et al. (2020)
Mg16820	*Meloidogyne*	Secretion in the apoplast and cytoplast of the host cell, and caused the suppression of cell death	*N. benthamiana*	Naalden et al. (2018)
Ubiquitin- proteins	Globodera	Modulate plant defense by cleaving extension peptide that regulate defense signaling	*N. benthamiana*	Chronis et al. (2013)
MgGPP	*Meloidogyne*	Posttranslational modulation in transport system in endoplasmic reticulum and nucleus	*Oryza sativa*	Chen et al. (2017)
28B03	*Heterodera*	Inactivate a stress-response kinase resulting in the blockage of kinase signaling leading to the phosphorylation of a host syntaxin protein that had been shown to condition the release of antimicrobial proteins into the apoplastic region	*N. benthamiana*	Kalde et al. (2007)
10A06	*Heterodera*	Interfere plant defense by targeting spermidine production, thus, reducing polyamine enzymatic activities and enhancing spermidine contents. This leads to an elevation of antioxidant activities in the plants	*Arabidopsis*	Hewezi et al. (2010)
MO237	*M. graminicola*	Suppresses host immunity mechanism by regulating defense gene expression, ROS, and callose deposition. Binding to pathogenesis-related proteins	*Oryza sativa*	Chen et al. (2018)

Albeit the nematode effectors mentioned above are released and secreted through oesophagal glands and stylet, many other nematode effectors can be secreted from various other organs or tissues (Hewezi, 2015). Recently, an interesting example is of the effectors of *Meloidogyne* (MIF like) released from the hypodermis of the nematode. These nematode effectors interacted with the annexin of the host plant. They suppressed the programmed cell death induced by various stimuli, and hence, the interaction is instrumental in impeding the plant’s defense responses. Mainly, MIF-2 possessed enhanced defense-repressing potentials like suppression of cell death and protection against reactive oxygen species. *G. rostochiensis* (potato cyst nematode) releases an allergin-like venomous protein in tomato plants, which particularly impedes the protease (papain-like cysteine Rcr3) in the apoplast and hence enhances the susceptibility to infection caused by nematodes (Rosso et al., 2011; Gul et al., 2022). Since the PPNs release protease along with vast amounts of enzymes responsible for degrading the host plant cell wall during the invasion process, both the enzymes and protein proteases might have a role in mediating the defense response of the host plant triggered by degradation of the plant cell walls (Thorpe et al., 2014).

## 6 Plant defensive metabolites as part of plant immunity and their action mechanism against nematode

Immunity in the host plants might have been enhanced by releasing the metabolites that play a role in repelling the PPNs. Several research findings revealed that ethylene or the ethylene-responsive pathway products repelled RKNs (Costa et al., 2020; Islam et al., 2024). The roots of mutants devoid of ethylene signaling enticed more nematodes than the wild type. Similarly, *Arabidopsis* plants with impeded ethylene synthesis also enticed more *M. hapla*, whereas the other roots of mutants where there was over-expression of ethylene lured very few nematodes (Piya et al., 2019; Bi et al., 2024). However, in the case of cyst nematode, the correlation between ethylene synthesis and the number of nematodes attacking the root system was not very clear. Soybean roots and *Arabidopsis*, with the application of ethylene inhibitors, enticed a higher number of nematodes (*H. glycines* soybean cyst nematode), and a significant number of nematodes were able to enter the roots of the plants in which synthesis of ethylene was restricted (Hu et al., 2017; Zhang et al., 2024).

The roots of mutant plants of Arabidopsis that were insensitive to ethylene were found highly attractive to *H. glycines* compared to their wild counterpart (Hu et al., 2020). Even at present, particular compounds have been shown to fend off only one nematode taxon in the case of a single species of host plant. Hence, it is very unlikely and premature to withdraw any valid conclusions that which particular repellents will most effectively repulse various PPN species. A few phytometabolites that effectively repel PPNs in some experiments without involving host plants could be tested for further validation. As an example, thymol derived from pepper (*Capsicum annum*) roots, either alone or in combination with other volatiles from the origins of pepper, triggered antagonistic chemotactic movement against cyst nematodes, root-knot nematodes as well as stubby root nematodes. A few of the flavonoids also demonstrated repulsion to PPNs; however, the impact of these compounds appeared to be highly species-dependent. Kaempferol, myricetin and quercetin flavonoids turned back *M. incognita* and *Radopholus similis*; however, no effect was observed in the case of *Pratylenchus penetrans*. Flavonoids like daidzein, genistein, and luteolin fended off *R. similis*, and there was no impact on *P. penetrans* and *M. incognita* (Chin et al., 2018; Detcharoenyos et al., 2024).

Several plant taxa, such as Brassicaceae and Tagetes, are known to produce and release compounds that repel nematodes. Plants known to have enhanced nematode inhibitory or nematicidal contents included as a sanitation practice have attracted significant research and are being in practice. Moreover, plant-derived nematicidal purified compounds could prove an effective control strategy against nematodes (Zanón et al., 2016). The precursor of sulfur amino acid present in the cytoplasm of Allium species gets degraded upon the destruction of cells by employing the alliinase enzyme into a novel volatile compound named DMDS (dimethyl disulfide) (Chiuta et al., 2021). DMDS in purified form destroyed J2s and decreased the masses of eggs and the formation of galls in the roots of tomato upon infection induced by *M. incognita* (Silva et al., 2019; Silva et al., 2022; Akhtar et al., 2023).

The most frequently investigated groups of secondary defense metabolites of plants are glucosinolates. In case of cellular destruction such as wounding produced by nematodes, myrosinases (endogenous enzymes) hydrolyzed the thioglucosidic linkages and resulted in the release of compounds (such as thiocyanate, isothiocyanate, epithionitrile, nitrile, and oxazolidine-2-thione) which are active against pathogens and herbivores (Santolamazza-Carbone et al., 2014). In its pure form, glucosinates from Brassicaceae exhibited no toxicity against second-stage juveniles of cyst nematode (*H. schachtii*); however, upon hydrolysis with enzymes, the resulting products were observed to be highly toxic to nematodes. Few other researches also demonstrated that glucosinolates are effective against cyst nematodes (*G. rostochiensis*) only if myrosinase is present (Eugui et al., 2022).

Secondary metabolites such as PAs (Pyrrolizidine alkaloids) are found in various species of host plants and were demonstrated to be lethal against RKNs (*M. incognita*; *P. penetrans*, and *H. schachtii*). Bioassay results revealed that exudates of Tall fescue roots containing PA had nematicidal efficacy against second-stage juveniles of *P. scribneri*. Similarly, *Senecio bicolour* and *Ageratum houstonianum* containing PA restricted the reproduction in *M. hapla*; however, reproduction occurred in *M. hapla* in the case of other species containing PA (Vestergaard et al., 2019). In marigold plants (*Tagetes* spp.), *α*-terthienyl, the widely studied nematicidal compound, is abundant (Hamaguchi et al., 2019). It was demonstrated under *in vitro* conditions that the exudates of marigold roots (*T. patula* cv. Single gold) decreased the population of *M. chitwood* and inhibited the reproduction of *P. penetrans* completely (Nježi et al., 2014). There might be some other bioactive compounds present in *Tagetes* that might have a role in the suppression of nematodes. However, there is no point in doubt about the potential of α-terthienyl as an efficient nematicidal compound (Hamaguchi et al., 2019).

Exudates of roots belonging to the group benzoxazinoids, including DIMBOA (2, 4-dihydroxy-7-methoxy-2H-1,4-benzoxazin-3(4H)-one), primarily released in many cereals including rye, have been demonstrated to exhibit toxicity against different stages of *Xiphinema americanum* (American dagger nematode) (Sikder et al., 2021). Cultivars of rye having enhanced concentrations of methoxy-substituted benzoxazinoids in their roots were shown to have a deficient number of eggs of *M. incognita*. Therefore, it was recommended to incorporate such cultivars in the soil as green manure to protect against RKNs (Frew et al., 2018). During a trial in the greenhouse, *M. incognita*-infested soil, when treated with 2,4-Dihydroxy-2H-1,4-benzoxazin-3(4H)-one (DIBOA) at 1.1 to 18 ug/g of the dry soil resulted in a significant decrease in the number of eggs of RKNs in cucumber roots mainly when applied at higher concentrations (Meyer et al., 2009).

Gene expression was impacted by applying plant metabolites in *M. incognita* second-stage juveniles before actually coming in contact with roots and penetrating them. Within 1 h of application, 63 candidate nematode genes were identified to be affected with variable levels of expression on exposure to plant root exudates, thus confirming the hypothesis that application of root exudates significantly impacts the gene expression in *M. incognita* (Teillet et al., 2013) Plant exudates have been demonstrated to modulate the gene expression responsible for encoding the cell wall degradation enzymes in nematodes that are used to break this physical barrier (Bell et al., 2019). Plant metabolites triggering the hatching of the cyst nematode eggs involve gene activation by exudates in the dormant juveniles (cyst nematodes) (Kang et al., 2018). It was exhibited in research studies on root lesions, root knots and cyst nematodes that exudates of plant roots affect the expression of genes at early pre-parasitic phases of nematodes; however, it is yet to be disclosed which components of the exudates are involved in regulating the gene expression (Ochola et al., 2021). We had very little information about the molecular reactions triggered by repellents, attractants and toxic compounds in the body of nematodes; however, it is documented in numerous studies that such exudates modulate the gene expression, including flp genes. These genes express FMRFamide-like proteins, a diversified neuropeptide group responsible for feeding behavior, mobility behavior, and reproduction in nematodes and thus have a vital role in the chemotaxis of nematodes (Kumari et al., 2017; Peymen et al., 2014).

In *Chrysanthemum coronarium* (crown daisy), reduced lauric acid concentrations in exudates of roots exhibited attraction of RKNs (*M. incognita*), whereas in the case of roots having enhanced lauric acid (4.0 mM) repelled the nematode. This behavior was most probably triggered by the amount of lauric acid that modulated the expression of the Mi-flip-18 gene (Dong et al., 2014). Furthermore, linoleic acid and palmitic acid are two other compounds produced by the roots of castor oil plants that also exhibited a repelling effect against *M. incognita* and restricted the gene expression of Mi-mpk-1 (mitogen-activated protein kinase) and Mi-flip-18 genes in a manner dependent on the concentration of exudates (Dong et al., 2018). As it is established that the *α*-terthienyl derived from the marigold plants possesses nematicidal potential in the soil, current research explored the molecular action of α-terthienyl with no light activation. It was demonstrated that this chemical is also effective under dark conditions although the efficacy is more when photoactivated. It was also shown that α-terthienyl a chemical that triggers oxidative stress and hence efficiently diffuses into the hypodermis of the nematode and represses sod-1 (superoxide dismutase) and gst-4 (glutathione S-transferase) expression of genes. It exhibited limited superoxide dismutase and glutathione S-transferase production, vital for defense responses against nematodes (Hamaguchi et al., 2019).

## 7 Conclusion

Plant pathogens, including plant parasitic nematodes, evolved different strategies to perceive and respond to these host plant defenses through their nervous system, which helps them to escape, avoid, or neutralize the host plant defense systems. To develop an effective management strategy, it is crucial to understand the mechanism by which the nematode suppresses the host defense. We explored the information on the modulation of host defenses by nematodes employing diverse approaches to host defense suppression. The role of nematode effectors in suppressing host immunity was discussed in detail. The insights into how plants responded to the nematode infections, including the release of metabolites, were given. The discussion provided in this review serves as a comprehensive resource guiding future studies in host-nematode interactions. Future studies could delve deeper into the molecular crosstalk between plants, nematodes, and other microbes. Understanding the rhizosphere microbiome will provide insights into how plant-associated microbes can aid in defense against nematode infections. Future research could also explore how microbiome engineering could support plant health and resistance against parasitic nematodes.
